# Case Report: Whole-exome sequencing revealed a de novo variant in *SETBP1* gene in a Chinese family with developmental delay

**DOI:** 10.3389/fgene.2025.1637931

**Published:** 2025-11-07

**Authors:** Junlin Pan, Yan Zhang, Jinwei Hou, Na Shi, Huiling Qu, Longhuan Jiang, Haiping Liu

**Affiliations:** Department of Reproductive Medicine, The 960th Hospital of the PLA Joint Logistics Support Force, Jinan, China

**Keywords:** whole-exome sequencing, SETBP1, developmental delay, pathogenic, case report

## Abstract

**Background:**

This study aims to characterize the potential genetic etiologies in children with developmental delay through whole-exome sequencing (WES) providing assistance for clinical diagnosis, genetic counseling, and reproductive guidance.

**Methods:**

WES was performed on the proband, followed by Sanger sequencing validation of the identified variant in the parents.

**Results:**

The proband exhibits global developmental delay, including impaired motor and language development, reduced spontaneous speech, poor coordination, and attention deficits. WES revealed a heterozygous nonsense variant in SETBP1 (c.1630C>T, p.Arg544Ter), which was confirmed as *de novo* by Sanger sequencing. This variant was classified as pathogenic according to American College of Medical Genetics and Genomics (ACMG) guidelines. The patient was subsequently diagnosed with intellectual disability, autosomal dominant 29 (MRD29).

**Conclusion:**

The *de novo* SETBP1 p.Arg544Ter variant was identified as the underlying genetic cause in this case. Our findings underscore the importance of early genetic testing in children with developmental delay to enable precise diagnosis, informed genetic counseling, and evidence-based reproductive planning.

## Introduction

SET-binding protein 1 (*SETBP1*) is a dosage-sensitive gene located at 18q12.3, encoding a regulatory protein critical for human brain development (Bellini et al., 2021; Cardo et al., 2023). As a transcription factor, SETBP1 participates in cell cycle regulation, modulates gene expression by influencing chromatin accessibility, and plays a role in transcriptional regulation (Antonyan and Ernst, 2022; Kohyanagi and Ohama, 2023). Gain-of-function (GoF) variants typically lead to abnormal enhancement of protein function, whereas loss-of-function (LoF) variants result in diminished or absent protein activity. These two types of *SETBP1* variants differ in disease mechanisms and clinical manifestations (Shaw et al., 2024; Jansen et al., 2021; Morgan et al., 2021).

It is known that the SETBP1 gene mutation that usually causes Schinzel-Giedion midface retraction syndrome is a GOF mutation. The LOF mutation is called SETBP1 haploinsufficiency disorder through the haploinsufficiency mechanism, and is also named as intellectual disability, autosomal dominant 29 (MRD29, OMIM #616078). The main characteristics of the disease include motor retardation, language retardation, intellectual disability and behavioral problems. Due to the phenotypic heterogeneity of MRD29, there is currently no unified diagnostic standard.

With the widespread clinical application of next-generation sequencing technology, whole-exome sequencing (WES) has now become a routine diagnostic tool for identifying the etiology of intellectual disability, developmental delay, and epilepsy in pediatric patients (Qiao et al., 2024; Lan et al., 2025; Brea-Fernández et al., 2022). This study reports a confirmed case of MRD29 resulting from a *de novo SETBP1* mutation, identified via whole-exome sequencing. We analyzed the genetic factors and clinical phenotypic characteristics associated with this mutation, providing guidance for genetic counseling and future reproductive decisions for the affected family.

## Materials and methods

### Patients

The proband is a 5-year-old girl who is the eldest child in her family. Her parents both are 34-year-old Chinese individuals, non-consanguineous, and in good health with no family history of genetic diseases. The mother’s obstetric history was G2P2, and the second child (the proband’s younger sister) was healthy with no abnormalities detected upon developmental assessment. The proband was born full-term via spontaneous vaginal delivery with a birth weight of 3,200 g and length of 51 cm. After birth, the proband was able to hold her head up at 4 months, sit at 7 months, stand at 7 months, walk at 18 months, say single words at 24 months. At the age of 4 years, the child shows delays in motor and language development, limited spontaneous speech, poor motor coordination, and impaired attention. Physical examination at age 5 revealed the following: height 110 cm (−1 SD), weight 18 kg (>-1 SD), and head circumference 49.8 cm (>-1 SD). The proband exhibited normal facial features, normal visual and hearing acuity without strabismus. Examination of the chest, heart, and abdomen revealed no significant abnormalities. Neurological examination showed poor fine motor skills, but symmetric tendon reflexes, negative pathological reflexes, a stable gait, and an absence of cerebellar ataxia. There was no history of epileptic seizures or behavioral abnormalities. Ancillary investigations, including blood myocardial enzyme spectrum, bone age, brain MRI, and electromyography, were all within normal limits. Liver and kidney function tests and essential metabolic screening also yielded no abnormal findings. GESELL developmental assessment gross motor skills, personal-social skills, auditory language, hand-eye coordination, and visual expression are all behind those of children of the same age. Wechsler Intelligence Scale assessment revealed a full-scale IQ score of 87. The proband was diagnosed with global developmental delay by the local hospital. To prevent the recurrence of similarly affected offspring, the parents underwent genetic counseling at our reproductive medicine center. To further clarify the underlying genetic etiology, We performed WES on the proband’s peripheral blood sample, followed by Sanger sequencing validation with blood samples from the proband’s parents and younger sister.

### WES and data analysis

Genomic DNA was extracted from the proband’s peripheral blood using the QIAamp DNA Blood Kit (QIAGEN) following standard protocols. The quality and quantity of DNA sample were assessed by 1% agarose gel electrophoresis, NanoPhotometer (IMPLEN, CA,United States) and Qubit^®^ 3.0 Flurometer (Life Technologies, CA, United States). Subsequent DNA libraries were constructed following the standard Illumina protocol. Exome capture was performed using the IDT xGen Exome Research Panel v1.0, and sequencing was conducted on the Illumina NovaSeq 6,000 platform. After processing the raw data against the GRCh37/hg19 human reference genome, the final quality metrics demonstrated the data’s suitability for variant calling. Specifically, each sample yielded over 12 GB of data, with a base call quality (Q30) above 90% and a mean target coverage of 100X. Moreover, 97% of the target regions were covered at a minimum depth of 20X. These metrics confirm that the WES data are of high quality and adequate for reliable downstream DNA variant analysis. Variant calling was performed with the GATK Toolkit, and annotation was conducted using ANNOVAR. High-frequency variants (minor allele frequency >0.01) were excluded using public databases (1,000 Genomes (accessed on 25 August 2023), ESP (accessed on 6 September 2023) and Chinamap database (accessed on 6 September 2023). The pathogenicity and conservation predictions of candidate variants were subsequently evaluated using multiple bioinformatics tools: OMIM, ClinVar, HGMD, PolyPhen-2, SIFT, MutationTaster, PROVEAN, CADD, Revel and SpliceAI. Candidate mutations were selected based on clinical phenotype and classified according to the American College of Medical Genetics and Genomics (ACMG) guidelines. To validate the candidate variant detected by WES, Sanger sequencing was performed on the proband and her parents. Additionally, the three-dimensional structures of wild-type and mutant SETBP1 proteins were predicted using the SWISS-MODEL online database (https://swissmodel.expasy.org/).

## Results

### Genetic findings

**FIGURE 1:**
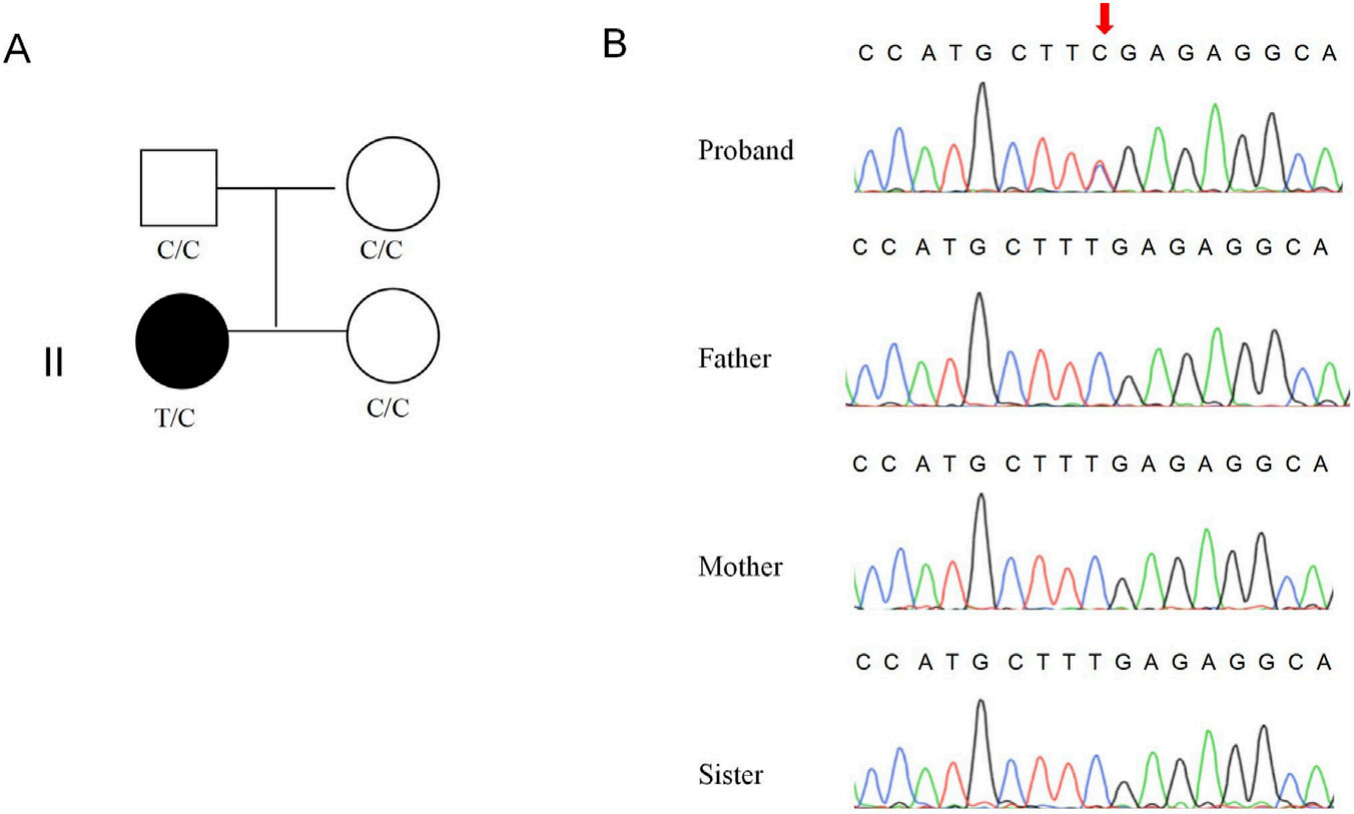
Genetic information of proband’s family. **(A)** Pedigree of the proband’s family. **(B)** Consequence of Sanger sequencing.

WES analysis identified a heterozygous nonsense variant (c.1630C>T, p.Arg544Ter) in the *SETBP1* gene (NM_015559.3) in the proband. This variant has been classified as pathogenic in both ClinVar and HGMD databases. To validate this finding, we performed Sanger sequencing analysis on the proband and her unaffected parents and younger sister. The Sanger sequencing validation results demonstrated that the c.1630C>T variant was exclusively identified in the proband (Figures 1A,B) and was undetectable in either unaffected parents and younger sister, confirming its status as a *de novo* mutation. In accordance with updated ACMG guidelines, the SETBP1 c.1630C>T variant was classified as “Pathogenic” based on the following evidence criteria: PVS1 (null variant in a gene where LoF is a known disease mechanism) + PM6 (*de novo* variant without familial segregation confirmation) + PS4_Supporting (The variant is more frequent in the affected cohort than in the control cohort) + PM2_Supporting (absent from controls in large population databases).

### Evolutionary conservation and protein structure prediction analysis

**FIGURE 2:**
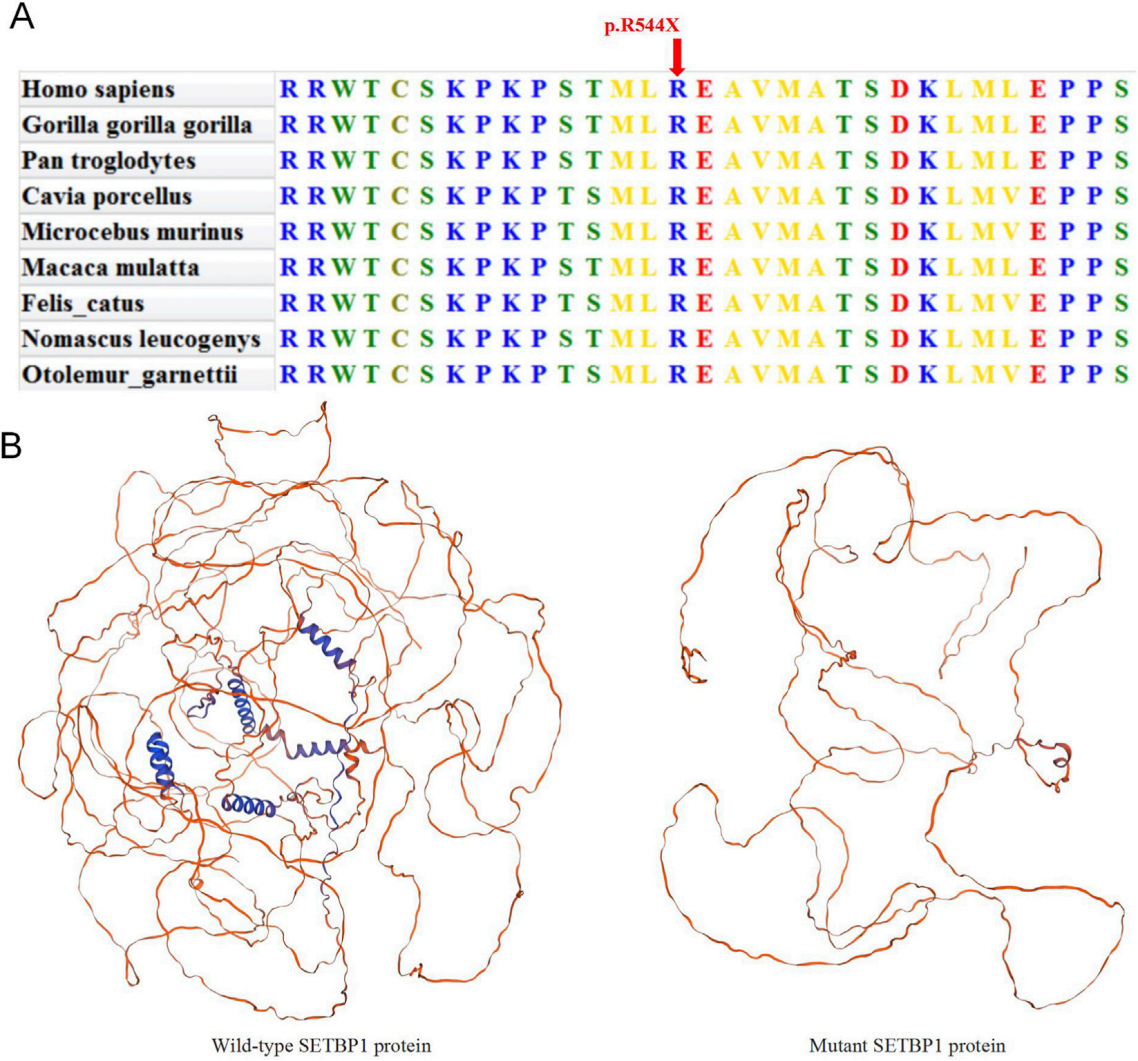
Predicting the impact of c.1630C>T variation on SETBP1 protein’s structure. **(A)** Conservation analysis among different species. **(B)** The protein structure of SETBP1 is depicted with and without the mutant variations.

**FIGURE 3:**
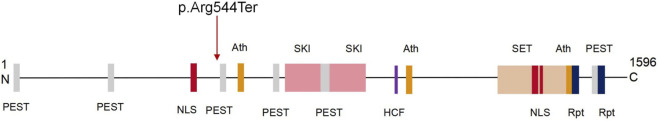
Schematic representation of SETBP1 protein.

Multiple sequence alignment of the SETBP1 gene across nine species revealed that the amino acid affected by the c.1630C>T variant is highly conserved (Figure 2A). The *SETBP1* protein sequence contains three AT-hook domains (Ath), a SKI homologous region, a HCF1-binding motif (HCF), a SET-binding domain (SET), three bipartite NLS motifs, six PEST sequences and two repeat domain (Rpt). Using Swiss-Model for protein tertiary structure prediction (Figure 2B), this variant leads to a nonsense mutation at nucleotide 1,630, where cytosine (C) is replaced by thymine (T), resulting in premature translation termination. Consequently, the protein structure beyond amino acid 544 is entirely lost, with only the two PEST and NLS motifs remaining to confer partial function, and all other domains abolished (Figure 3). This extensive damage is predicted to severely compromise the overall function of the SETBP1 protein.

## Discussion

This study reports a case of developmental delay caused by a pathogenic *SETBP1* mutation (c.1630C>T, p.Arg544Ter), which was identified through WES and validated by familial segregation analysis. Importantly, this study provides additional evidence that *SETBP1* gene mutations may be associated with developmental delay, suggesting new directions for investigating the genetic basis of developmental disorders.

*SETBP1* is located on chromosome 18q12.3 and primarily expressed in the nucleus. This gene encodes a 170 kDa protein containing several predicted functional domains: a SKI homologous region, a SET-binding domain, three AT-hooks, and repetitive domains. Traditionally, *SETBP1* mutations were recognized as the genetic cause of Schinzel-Giedion syndrome, a condition characterized by multiple severe congenital malformations (Yang et al., 2022; Zheng et al., 2024). However, in 2011, the identification of microdeletions encompassing SETBP1 in two patients with mild developmental delay led researchers to propose that haploinsufficiency of SETBP1 might underlie milder developmental impairments (Filges et al., 2011). This hypothesis was subsequently validated through further investigations by Coe et al. (2014). Ultimately, Jansen et al. established the diagnostic term “SETBP1 haploinsufficiency disorder” and conducted systematic studies to characterize its clinical phenotype (Jansen et al., 2021).

In previously reported cases of MRD29, the majority of pathogenic variants were frameshift or nonsense mutations, predominantly occurring *de novo* (Wang L. et al., 2023; Vrkić Boban et al., 2020; Wang H. et al., 2023; Miolo et al., 2024). Most patients with MRD29 exhibit language and motor developmental delays accompanied by intellectual disability, while some present with additional features including inattention, visual impairment, epilepsy, and facial dysmorphism (Leonardi et al., 2020; Eising et al., 2019; Hamdan et al., 2014). A subset of MRD29 patients may also demonstrate hearing loss, hypotonia, or autistic behaviors. The results of this study showed that this patient had a c.1630C > T mutation of *SETBP1*, which was highly matched with the characteristic clinical manifestations of MRD29, including significant delay in motor and language development (especially in reduced spontaneous speech), impaired motor coordination, attention deficit and stereotyped behavior. Based on this genotype-phenotype correlation, the patient was finally diagnosed with MRD29.

MRD29 exhibits significant clinical heterogeneity, and no clear genotype-phenotype correlation has been established to date. To date, a total of four cases (including the present study) carrying the *SETBP1* gene c.1630C>T mutation have been documented, all of which were confirmed as *de novo* variants (Table 1). The mutation exhibits a broad phenotypic spectrum with varying severity. One case presented solely with intellectual disability (Brea-Fernández et al., 2022), another exhibited intellectual disability accompanied by motor developmental delay (Morgan et al., 2021), while a more severe case manifested a combination of intellectual disability, motor developmental delay, attention deficit, facial dysmorphism, and hypotonia (Jansen et al., 2021). The *SETBP1* hotspot variant c.1873C>T (p.Arg625Ter), although reported in 6 confirmed cases, showed significant phenotypic variation in affected individuals (Jansen et al., 2021; Morgan et al., 2021; Coe et al., 2014). In a study by Wang et al. involving a non-consanguineous family, four members carrying the identical c.942_943insGT (p.Asp316TrpfsTer28) variant exhibited distinct clinical manifestations: while the affected mother presented with auditory abnormalities and white matter lesions, her affected daughter showed none of these features (Wang H. et al., 2023). This observation strongly suggests that identical variants within the same pedigree can produce differential phenotypic expression. Consequently, comprehensive screening of *SETBP1* mutation patterns through WES significantly enhances diagnostic accuracy for MRD29, thereby facilitating timely clinical interventions.

**TABLE 1:** Major clinical phenotypes reported in patients with c.1630C>T mutation in *SETBP1* gen**e**.

Case	Gender	Age	Motor developmental delay	Language developmental delay	Intellectual disability	Visual impairment	Attention deficit	Epilepsy	Facial dysmorphism	Hearing impairment	Hypotonia	Allele origin	References
Case 1	Female	3 years old	+	+	_	_	+	_	+	_	+	*de novo*	Jansen et al. (2021)
Case 2	Female	3 years old and 7 months old	+	+	NA	NA	_	NA	_	_	NA	*de novo*	Morgan et al. (2021)
Case 3	Male	NA	NA	NA	+	NA	NA	NA	NA	NA	NA	*de novo*	Brea-Fernández et al. (2022)
Case 4	Female	4 years old	+	+	_	_	+	_	_	_	_	*de novo*	This Study

+, feature present; −, feature absent; NA, feature not reported.

SETBP1 protein is widely expressed in human tissues, and its expression reaches a peak during fetal brain development, which is a critical period for nerve cell proliferation and migration to specific brain regions. Given the important role of SETBP1 in this developmental window, genetic variation in this gene may lead to neurodevelopmental disorders. Although this study has provided significant evidence suggesting a potential association between *SETBP1* gene variants and MRD29, it must be acknowledged that this study has certain limitations. First, as a single-case report, the generalizability of our findings and conclusions is inherently limited. Second, although we confirmed the pathogenic variant through WES and Sanger sequencing, the sensitivity of these methods restricts their ability to detect low-level somatic mosaicism. Consequently, we cannot entirely rule out the possibility of mosaicism as a potential influencing factor. Finally, our study focused on genetic sequence analysis and did not investigate the aspects of epigenetic regulation. Future studies that expand the sample size, employ more sensitive techniques for mosaicism detection, and integrate multi-omics analyses to explore epigenetic mechanisms will contribute to a more comprehensive understanding of the pathogenic mechanisms underlying SETBP1-related disorders.

This study reports a pediatric case of developmental delay in which WES identified a heterozygous variant (c.1630C>T, p.Arg544Ter) in the *SETBP1* gene, subsequently confirmed as a *de novo* mutation by Sanger sequencing, leading to a definitive diagnosis of MRD29. Due to the highly heterogeneous clinical phenotype of MRD29, clinical diagnosis is difficult, and genetic testing has emerged as the definitive diagnostic approach. For children with developmental delay, early genetic testing is recommended to clarify the diagnosis, which not only helps to develop individualized diagnosis and treatment programs, but also provides guidance for genetic counseling and future reproductive planning.

## Data Availability

The data generated and analyzed in this study are not publicly available due to ethical restrictions and institutional policies regarding patient confidentiality. The informed consent obtained from participants does not allow for public data sharing, in compliance with the approved protocol by the ethics committee. However, the data may be made available to qualified researchers upon reasonable request and with appropriate ethical approvals. Further inquiries can be directed to the corresponding author at haipingliu960@163.com.
